# Fear Stress During Pregnancy Affects Placental m6A-Modifying Enzyme Expression and Epigenetic Modification Levels

**DOI:** 10.3389/fgene.2022.927615

**Published:** 2022-06-22

**Authors:** Qiyang Wang, Mingmin Pan, Tong Zhang, Yu Jiang, Peiyuan Zhao, Xihong Liu, Anqi Gao, Liping Yang, Junlin Hou

**Affiliations:** Medicine College, Henan University of Chinese Medicine, Zhengzhou, China

**Keywords:** fear stress, placenta, N6-methyladenosine, methylation enzymes, demethylase, double aberrant genes

## Abstract

As the hub connecting mother and offspring, the placenta’s normal development is vital for fetal growth. Fear stress can cause some structural alterations in the placenta and affect placental development and function. N6-methyladenosine (m6A) is the most common mRNA modification and is involved in regulating the development of the placenta and embryo. There are no reports on the potential role of m6A modification in placental damage caused by fear stress during pregnancy. In this study, we demonstrated that fear stress during pregnancy increases the levels of methylated enzymes (METTL3, METTL14, and WTAP), decreases the levels of demethylase FTO, and increases the overall methylation levels in the placenta of pregnant rats. MeRIP-seq data analysis revealed 22,010 m6A peaks associated with 12,219 genes in the placenta of the model and 21,060 m6A peaks associated with 11,730 genes in the placenta of the control. The peaks were mainly concentrated in the coding region and the 3ʹ untranslated region. In addition, 50 genes with abnormal modification and expression (double aberrant genes) were screened out by combining MeRIP-seq and RNA-seq data. *Mefv*, *Erbb2*, and *Cgas* were selected from 50 double aberrant genes, and MeRIP-qPCR and real-time quantitative polymerase chain reaction were used to verify their modification and expression levels. Our findings suggest that m6A modifications play an important role in placental dysfunction induced by fear stress during pregnancy.

## Introduction

Increasing stress and trauma due to factors such as competition and emergencies have led to a significant increase in psychologically-induced illnesses (Turner et al., 2020). Pregnant women are especially susceptible to psychological problems caused by external stimuli during pregnancy (Smith et al., 2020). Fear stress causes significant physical and mental harm to pregnant women and dramatically increases the risk of fetogenic diseases and fetal malformations (Tsui et al., 2006; Glover and Capron, 2017; Mizrak Sahin and Kabakci, 2021). Therefore, the prevention and treatment of fetal diseases have become an urgent social issue.

The hypothalamic–pituitary–adrenal (HPA) axis has been the focus of most studies on the relationship between stress and fetal stunting. It is hypothesized that pregnancy stress can overactivate maternal HPA axis function, thereby resulting in increased glucocorticoid (GC) levels (Howland et al., 2017; Anifantaki et al., 2021; Rybnikova and Nalivaeva, 2021). Maternal GC overexpression causes alterations in intrauterine HPA axis programming in the offspring, thereby affecting the development and health of the offspring (Howland et al., 2017). However, it has also been suggested that the placenta is a promising programming vehicle (Montoya-Williams et al., 2018; Musillo et al., 2022). Recent studies have found that the genetic changes regulated by placental N6-methyladenosine (m6A) modifications play a pivotal role in fetal growth and preeclampsia (PE) development (Wang et al., 2020a; Wang et al., 2021).

M6A is the most prevalent, abundant, and highly conserved internal modification of eukaryotic mRNA. It is dynamically regulated by “writers” and “erasers,” influencing the destiny of transcripts through “readers.” (He et al., 2019; Zaccara et al., 2019). Most of the m6A methylation on the mRNA is installed by a writer complex consisting of the core subunits METTL3 and METTL14, and additional adaptor proteins, including WTAP, Virma, ZC3H13, Hakai, and RBM15/15B. Another writer, METTL16, can install m6A in the sequence of UAC (m6A) GAGAA at the top of the hairpin structure in the transcript MAT2A. Two erasers, FTO and ALKBH5, have been identified for m6A demethylation on mRNA. Reader proteins prefer binding to m6A-containing RNAs using different mechanisms. Proteins containing the YTH domain (YTHDF1-3, YTHDC1-2) directly recognize m6A methylation using a well-characterized YTH domain. RNA-binding proteins such as IGF2BP1-3 and FMR1 prefer m6A-containing RNAs through tandem common RNA-binding domains (RBDS) (Frye et al., 2018; Shi et al., 2019). One study revealed that fear stress could regulate the expression of m6A effectors in different regions of the brain, thereby affecting m6A modification levels (Widagdo et al., 2016; Walters et al., 2017; Engel et al., 2018). However, it is unknown whether fear stress triggers placental dysfunction by altering the levels of placental m6A effectors and m6A modification.

Herein, we studied the changes in methylation enzymes (METTL3, METTL14, and WTAP) and demethylases (FTO and ALKBH5) in the placenta caused by fear stress during pregnancy as well as m6A modification levels of total RNA in the placenta. All differentially modified and aberrantly expressed genes were screened using RNA-seq and MeRIP-seq. Accordingly, it was hypothesized that an imbalance in placental m6A modifications is the causal mechanism underlying placental damage induced by fear stress.

## Materials and Methods

### Animals

Wistar rats (*n* = 55; 35 females and 20 males; weight 260 ± 30 g) were purchased at 11 weeks of age from the Beijing Vital River Laboratory Animal Technology Co., Ltd. [License No.: SCXK (Beijing) 2016-0006; Animal Certificate No.: 11400700319215]. The rats were fed at the Experimental Animal Center of the Henan University of Chinese Medicine (Henan, China) and housed in sterile animal colonies under the following conditions: 25 ± 3°C temperature, 45 ± 5% humidity, and 12 h light/dark cycle. This study was conducted at Henan University of Chinese Medicine according to the guidelines of the National Institutes of Health Guide for the Care and Use of Laboratory Animals. The procedures were approved by the Animal Ethics Committee of Henan University of Chinese Medicine (permit number: DWLL2018030017).

### Pregnant Rat Model of Fear Stress

After 1 week of adaptive feeding, all the rats were subjected to a baseline test. First, female rats with consistent scores were selected. Next, 15 male rats with the consistent baseline scores were selected as mating rats, and the rest were used as electroshock rats. Finally, according to the 2:1 mating cage ratio, 24 pregnant female rats were randomly divided into control group and fear stress model group, with 12 rats in each group.

The fear stress model was established according to modified bystander electroshock method (Geng et al., 2019). In this method, a stimulus was applied once per day for 20 days. The electroshocked and model rats were placed in corresponding chambers of a homemade electroshock communication box at the same time every day. The electroshocked rats received electric shocks in the chamber, whereas the model rats received fear messages from the electroshocked rats via sight, sound, and smell stimuli from their position in the spectator chamber, thereby generating fear and corresponding psychological stress.

### Behavioral Tests

#### Open Field Test

The open field test was used to evaluate exploratory behavior, anxiety, and depression in animals. The rats were placed in an open black box (dimensions, 100 × 100 × 40 cm^3^). The bottom of the box was divided into 25 squares of equal size by white lines, and a quiet experimental environment was ensured. Each rat was lifted by grasping the tip of its tail at one-third of the root and was gently placed in the middle of the open field chamber. The number of times it crossed the grid (we scored one point for crossing one gridline with both hind limbs) or stood upright (we scored one point for lifting both forelimbs off the ground) within a 3-min period following 1 min of adaptation was recorded. Immediately after the rat was subjected to the test, we wiped the inner walls and bottom of the box with alcohol and ventilated the chamber for 2 min before performing the test on the next rat.

#### Fear Condition Test

The fear condition test was performed to evaluate the acquisition, extraction, and regression of fear memory in animals. On the day before the test, the rats were acclimatized by placing them in a scenario fear box for 2 min, followed by a stimulation procedure. This procedure consisted of 30 s of noise stimulation (1 Khz, 80 Db) interspersed with 2 s of light stimulation (310 Lux) and 2 s of electrical stimulation (0.5 mA), followed by 30 s of rest. Acclimatization comprised four cycles of this stimulation procedure, which lasted 6 min. The fear condition test was conducted 24 h later. The test time was 6 min, including 2 min of adaptation and 2 min of noise and light stimulation of the same intensity but without electrical stimulation. A computer automatically tracked the duration of rigidity and immobility in the rats as “freezing time,” which was used as an indicator to assess fear in the stress model rats.

### Placenta Collection

After the behavioral test, the animals were anesthetized using 2% pentobarbital (3 ml/kg). Blood was drawn from the abdominal aorta, and 24 pregnant rats were sacrificed by cervical dislocation. All the placentas were manually separated from the endometrium. The weight and diameter of the placentas in each litter were evaluated, and two placentas from each litter were randomly selected. The round tissue with a diameter of 5 mm around the umbilical cord of each placenta was taken out, and stored in a cryogenic refrigerator at −80°C for subsequent RNA and protein extraction. The remaining placenta was used for another experiment. All the above steps were completed in an aseptic environment, and the sample collection process was completed within 5 min of separating the placenta *in vitro*.

### Enzyme-Linked Immunosorbent Assay

The levels of ACTH, GC, and estriol in the serum of pregnant rats were determined using enzyme-linked immunosorbent assay (*n* = 12). All steps were conducted according to the manufacturer’s instructions: 1) the absorbance of each sample was measured at 450 nm, 2) the absorbance served as the vertical coordinate and a corresponding standard concentration served as a horizontal coordinate to plot a standard curve, and 3) the ACTH, GC, and estriol levels in each sample were calculated according to the regression equation of the standard curve.

### Histological Analyses of the Placentas

The placentas were fixed in 4% paraformaldehyde solution, dehydrated with a gradient solution of ethanol and water, and purified by xylene. Subsequently, they were paraffin-embedded and cut into 5-μm thick sections. The sections were stained with hematoxylin and eosin. Finally, micrographs were obtained using a light microscope (Nikon, E100, Japan).

### Western Blot Analysis

Six biological replicates were selected from each group, and RNA and protein were extracted for western blot analysis and real-time quantitative polymerase chain reaction (RT-qPCR). RIPA lysate (Beyotime Biotechnology, Shanghai, China) was used to extract total protein from the placental tissues. The total protein concentration was determined according to the instructions of the BCA kit. The total protein samples were separated via SDS-PAGE (stacking gel, 4%; separating gel, 8%). After electrophoresis, the separated protein bands were transferred to PVDF membranes. Subsequently, the membranes were blocked with a blocking solution containing 5% skimmed milk powder and TBST solution for 1 h. The membranes were then incubated overnight at 4°C with a primary antibody, e.g., anti-METTL3 or anti-GAPDH (1:1,000; Abcam, Cambridge, United Kingdom); anti-METTL14, anti-WTAP, or anti-FTO (1:1,000; CST, Boston, MA, United States); or anti-ALKBH5 (1:1,000; Novus Biologicals, Littleton, CO, United States). The membranes were then washed thrice for 10 min each with TBST. A secondary antibody [goat anti-rabbit IgG H&L (HRP), 1:10,000; rabbit anti-mouse IgG H&L (HRP), 1:10,000; Abcam, Cambridge, United Kingdom] was then added, and the membrane was incubated for 1 h. The membrane was subsequently washed thrice with TBST for 10 min. The blots were visualized using a Tanon 6,600 luminescence imaging workstation (Tanon, Shanghai, China) and optical density values were analyzed using Image Pro Plus 6.0 software. The protein levels were measured and expressed relative to the expression level of the internal reference protein GAPDH.

### RT-qPCR

Total RNA was extracted from the placental tissues using TRIzol reagent (Invitrogen, Carlsbad, CA, United States) according to the manufacturer’s instructions. RNA was reverse transcribed into cDNA using the iScript™ Advanced cDNA Synthesis Kit (Bio-Rad, Hercules, CA, United States). cDNA was extracted using the SoFast EvaGreen Supermix (Bio-Rad Laboratories, CA, United States), and RT-qPCR was performed using an ABI 7500 real-time fluorescence quantitative PCR instrument (ABI, CA, United States). The relative expression levels were calculated using the 2^−ΔΔCt^ method, which were then normalized against GAPDH mRNA levels. The specific primer sequences are shown in Table 1.

**TABLE 1:** Primers used for real-time quantitative polymerase chain reaction.

Primers	Sequence (5′→3′)	
*FTO*	Forward	5′-GTG​TGA​CAA​ATG​CCG​TGC​TT-3′
	Reverse	5′-TGC​TGT​GCT​GGT​AGA​GTT​CG-3′
*ALKBH5*	Forward	5′-ACG​GCC​TCA​GGA​CAT​CAA​AG-3′
	Reverse	5′-AAG​CAT​AGC​TGG​GTG​GCA​AT-3′
*METTL3*	Forward	5′-ATG​TGC​AGC​CCA​ACT​GGA​TT-3′
	Reverse	5′-CTG​TGC​TTA​AAC​CGG​GCA​AC-3′
*METTL14*	Forward	5′-CAC​CGC​TAC​CAG​TCT​TGG​AC-3′
	Reverse	5′-ATC​TGC​ACT​CTC​AGC​TCC​CA-3′
*WTAP*	Forward	5′-CCT​CGC​CTC​GTC​TCT​TCT​GG-3′
	Reverse	5′-GTC​ATC​TTG​TAC​CCC​GAG​ACG-3′
*Mefv*	Forward	5′-AGA​AAT​GCT​GGG​CTC​CGA​AAT-3′
	Reverse	5′-GAC​GGA​TGA​AGG​TAA​TCT​TGA​GG-3′
*Erbb2*	Forward	5′-TGG​AGG​AGT​GCC​GAG​TAT​GGA-3′
	Reverse	5′-AAT​GAT​GAA​TGT​CAC​CGG​GCT-3′
*Cgas*	Forward	5′-AGC​CAG​ACA​AGC​TAA​AGA​AGG​TG-3′
	Reverse	5′-GCA​GCA​GTT​GAT​CCA​CGA​CTT​TAT-3′
*GAPDH*	Forward	5′-TGA​TTC​TAC​CCA​CGG​CAA​GTT-3′
	Reverse	5′-TGA​TGG​GTT​TCC​CAT​TGA​TGA-3′

### m6A RNA Methylation Assay

Total RNA was extracted from each group of six samples. The EpiQuik m6A RNA Methylation Quantification Kit (Epigentek, United States) was used to assess the global m6A modification levels of the mRNA. Briefly, 200 ng of poly(A) purified RNA was added to each well, following which the relevant antibody was added to each well individually at the appropriate dilution. The OD450 of each well was measured. m6A modification levels were quantified from the standard curve and calculated.

### MeRIP-Seq and RNA-Seq

Sequencing was performed for three rats from each group. Poly(A) RNA was purified from 50 μg of total RNA using Dynabead Oligo (dT) 25-61005 (Thermo Fisher, CA, United States) in two purification rounds. Poly(A) RNA was then fragmented into small pieces using a Magnesium RNA Fragmentation Module (NEB, cat.e6150, United States) at 86°C for 7 min. A portion of the fragmented RNA was used as an RNA-seq library. Cleaved RNA fragments were then incubated for 2 h at 4°C with an m6A-specific antibody (No. 202003, Synaptic Systems, Germany) in IP buffer (50 mM Tris-HCl, 750 mM NaCl, and 0.5% Igepal CA-630). The IP RNA was reverse transcribed into cDNA using SuperScript™ II Reverse Transcriptase (Invitrogen, cat. 1896649, United States). The cDNA was then used to synthesize U-labeled second-stranded DNA using *Escherichia coli* DNA polymerase I (NEB, cat.m0209), RNase H (NEB, cat.m0297), and dUTP solution (Thermo Fisher, cat.R0133). An A base was then added to the blunt ends of each strand to prepare them for ligation to the indexed adapters. Each adapter contained a T-base overhang for ligating the adapter to the A-tailed fragmented DNA. Single- or dual-index adapters were ligated to the fragments, and size selection was performed using AMPureXP beads. The U-labeled second-stranded DNA was treated with heat-labile UDG enzyme (NEB, cat.m0280), and the ligated products were amplified using PCR at the following conditions: initial denaturation at 95°C for 3 min; eight cycles of denaturation at 98°C for 15 s, annealing at 60°C for 15 s, and extension at 72°C for 30 s; and final extension at 72°C for 5 min. The average insert size of the final cDNA library was 300 ± 50 bp. Finally, we performed 2 × 150 bp-paired-end sequencing (PE150) on Illumina Novaseq™ 6,000 (LC-Bio Technology Co., Ltd., Hangzhou, China) following the manufacturer’s recommended protocol.

### Gene-Specific m6A qPCR

The IP products were collected and reverse transcribed into cDNA according to the MeRIP-seq process described above. Gene-specific primers were then designed for qPCR-based quantification. The relative expression levels were calculated using the 2^−ΔΔCt^ method and were normalized against GAPDH mRNA expression levels. The primers used for PCR are presented in Table 2.

**TABLE 2:** Sequences of primers used for m6A-qPCR.

Primers	Sequence (5′→3′)	
*Mefv*	Forward	5′-AGG​AAT​GGA​ATG​AAT​AGG​AA-3′
	Reverse	5′-TGG​ACC​CCA​GTC​AGA​GTA​AC-3′
*Erbb2*	Forward	5′-ACC​CTG​AAT​ACT​TAG​TAC​CGA​G-3′
	Reverse	5′-GAG​TTC​TGG​TCC​CAG​TAA​TAG​AG-3′
*Cgas*	Forward	5′-GAT​GTT​CAT​GTA​ACC​CTG​GCT-3′
	Reverse	5′-TGC​TAT​CAA​GCA​TGG​TAG​CAC-3′

### Bioinformatics Analysis of MeRIP-Seq and RNA-Seq Data

**FIGURE 1:**
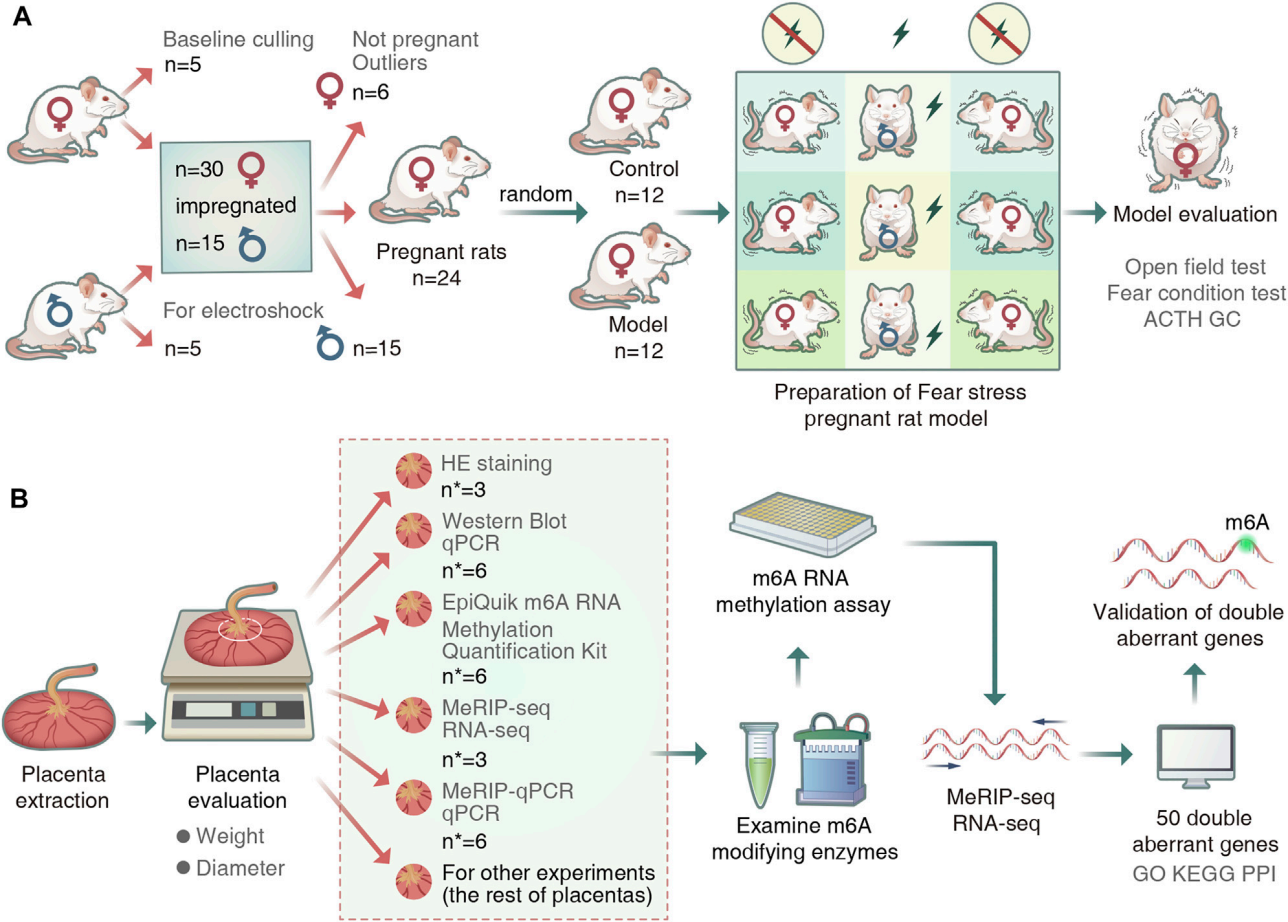
Experimental procedure. **(A)** Animal grouping and model preparation. (“*n*” represents the number of rats). **(B)**Schematic diagram of the experimental workflow. (“*n* ^∗^” represented the number of placentas).

We performed quality control of the raw data of placental samples using fastp (v0.19.4). We used the sequence alignment tool HISAT2 (v2.0.4) to compare the genome (v101) to reference sequences. Peak calling and gene difference peak analysis were performed using the exomePeak (v1.9.1) package for R (version 4.1.2). All peaks were annotated using ChIPseeker (v1.18.0). Finally, we performed motif analysis using MEME2 (v4.12.0) and HOMER (v4.1). Differential peaks and differentially expressed genes in the placental samples were identified if the sample showed a fold count of ≥1.5 and a *p*-value of <0.05. Gene enrichment analysis was performed based on Gene Ontology (GO) and Kyoto Encyclopedia of Genes and Genomes (KEGG) annotations. The overall experimental flow is shown in Figure 1.

### Statistical Analyses

All summary data were expressed as mean ± standard deviation, and all statistical tests were performed using SPSS version 21.0 (IBM SPSS, United States). Comparisons between two groups were performed using *Student’s t-test*, and comparisons of non-normally distributed data were performed using *Mann–Whitney U test*. For all tests, a *p*-value of <0.05 indicated statistical significance.

## Results

### Evaluation of the Pregnant Rat Models of Fear Stress

**FIGURE 2:**
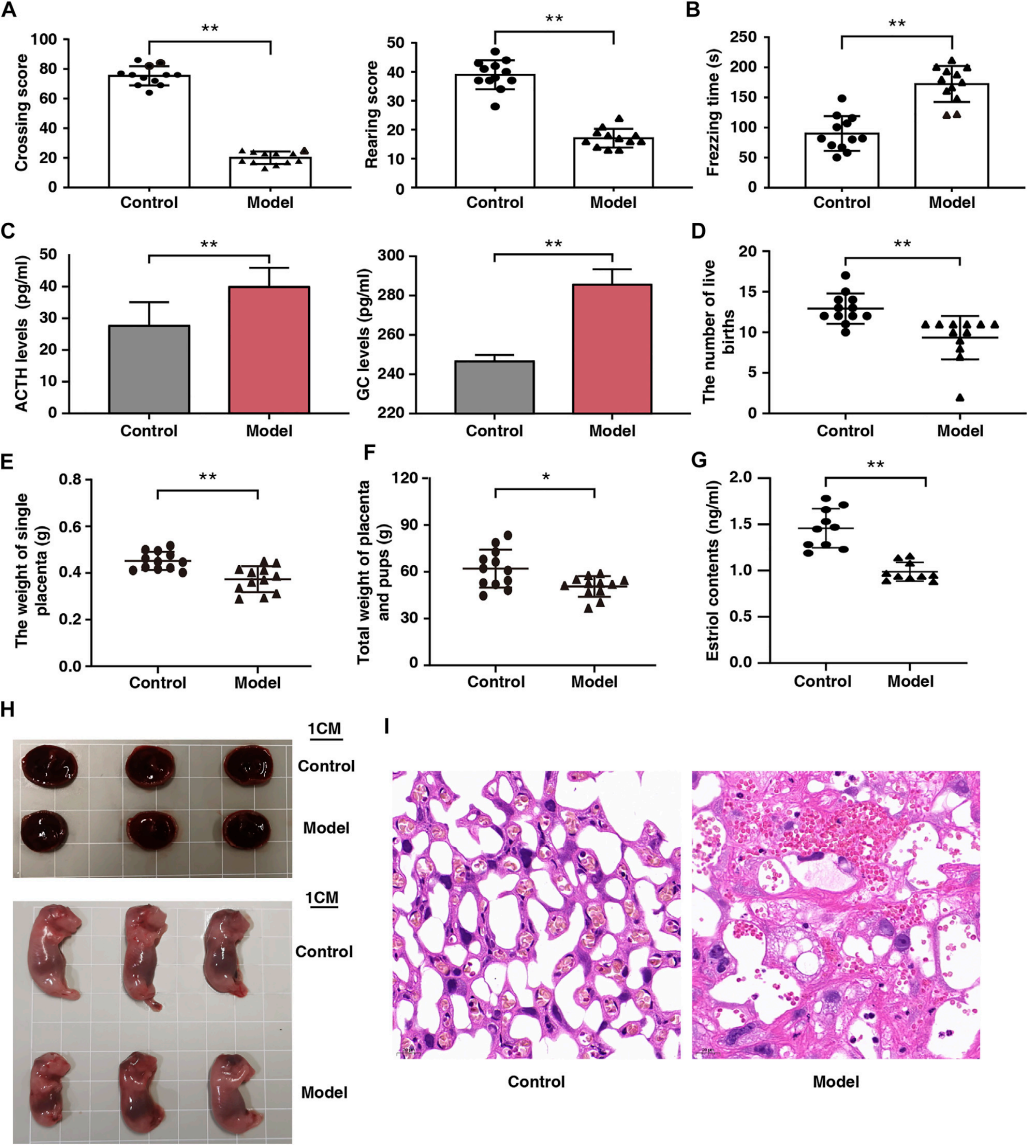
Effect of fear stress during pregnancy on the behavior and fetal outcomes of pregnant rats. **(A)** Crossing and rearing scores of pregnant rats in control and model groups (*n* = 12, ^**^ *p* < 0.01, ^**^ *p* < 0.01). **(B)** Freezing time of pregnant rats in control and model groups (*n* = 12, ^**^ *p* < 0.01). **(C)** ACTH and GC levels in control and model groups (*n* = 12, ^**^ *p* < 0.01, ^**^ *p* < 0.01). **(D)** The number of live birth of pregnant rats in control and model groups (*n* = 12, ^**^ *p* < 0.01). **(E)** The weight of single placenta of pregnant rats in model and control groups (*n* = 12, ^**^ *p* < 0.01). **(F)** Total weights of the placenta and pups of pregnant rats in control and model groups (*n* = 12, ^*^ *p* < 0.05). **(G)** The estriol levels of pregnant rats in control and model groups (*n* = 12, ^**^ *p* < 0.01). **(H)** Placenta and morphological results of offspring in control and model groups (Scale bars, 1 cm). **(I)** Placental sections obtained from pregnant rats (stained with hematoxylin and eosin; scale bar, 20 μm). ACTH, Adrenocorticotropic hormone; GC, Glucocorticoid.

Open field and fear condition tests were used to assess emotional changes in the pregnant rats. Compared with the control rats, horizontal and vertical scores were lower and freezing time was longer in the model rats (Figures 2A,B). In addition, there was a significant increase in ACTH and GC levels in the model rats (Figure 2C). These results indicate that the fear stress model was successfully established and could be used for further research.

### Reduced Placental Weight and Impaired Placental Function Due to Fear Stress

After fear stimulation, we observed that the live births of model rats had decreased by 28%; individual placenta weights, 20%; and total placenta and offspring weights, 19%. (Figures 2D–F). These findings indicate that fear stress can decrease placenta weight and offspring viability. We observed that serum estriol levels in the models were markedly reduced (Figure 2G), suggesting that fear stress impairs placental function.

### Histological Analysis of the Placenta of the Pregnant Rat Models of Fear Stress

Placental development was analyzed from the perspectives of gross morphology and histology. The placenta was smaller and the weight of the offsprings was lower in the model rats than in the control rats (Figure 2H). Histological analysis revealed that the placenta was tightly arranged with abundant vascularity and well-developed branching at the interface of the labyrinth layer in the control rats, whereas it showed extensive disorganization of the tissue structure, narrowed capillaries, and multiple vacuoles of varying sizes in the model rats (Figure 2I).

### Fear Stress Alters Placental m6A-Modifying Enzyme Expression

**FIGURE 3:**
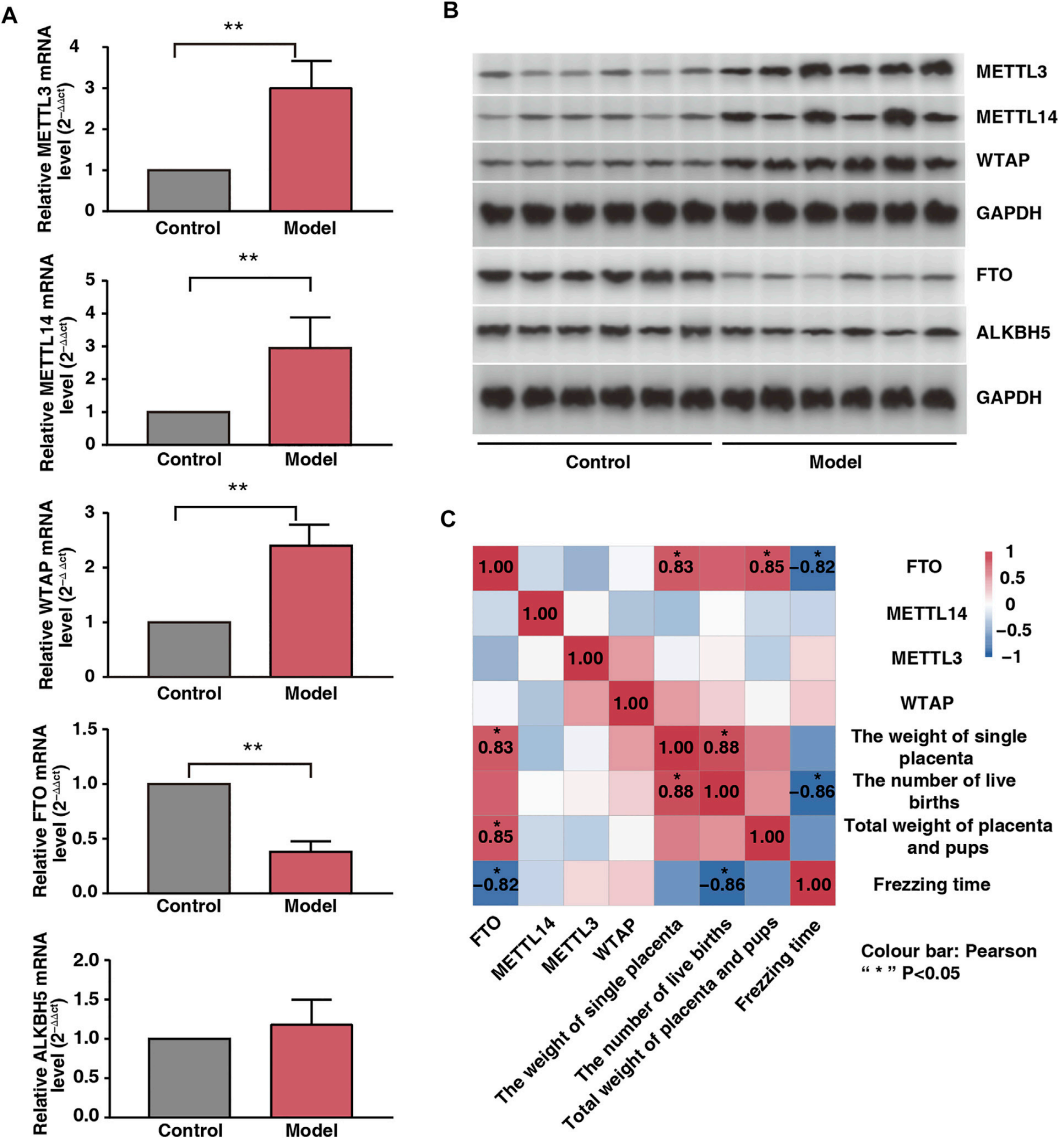
Effect of fear stress during pregnancy on placental methylase. **(A)** PCR analysis of enzymes (METTL3, METTL14, WTAP, FTO and ALKB5) in control and model groups (*n* ^*^ = 6, ^**^ *p* < 0.01, ^**^ *p* < 0.01, ^**^ *p* < 0.01, ^**^ *p* < 0.01, *p* > 0.05). **(B)** Western blot analysis of enzymes (METTL3, METTL14, WTAP, FTO and ALKB5) in control and model groups (*n* ^*^ = 6, ^**^ *p* < 0.01, ^**^ *p* < 0.01, ^**^ *p* < 0.01, ^**^ *p* < 0.01, *p* > 0.05). **(C)** Correlation analysis of four enzymes (METTL3, METTL14, WTAP, and FTO) with the emotions and fetal outcomes of pregnant rats.

To examine the placental expression of methylesterase, we randomly selected six placental tissue from each group. Our results showed that the mRNA expression levels of METTL3, METTL14, and WTAP were significantly upregulated; those of FTO were significantly downregulated; and those of ALKBH5 did not change significantly in the model rats (Figure 3A). The protein expression levels of METTL3, METTL14, WTAP, FTO, and ALKBH5 were consistent with their mRNA expression levels (Figure 3B). Freezing time is considered an effective indicator for evaluating animal fear. We assessed the correlation between the expression levels of the four enzymes and freezing time and found that FTO was negatively correlated with freezing time (*r* = −0.82, *p* < 0.05). In addition, FTO was positively correlated with total fetal weight and single placental weight (Figure 3C; *r* = 0.85 and *r* = 0.83, respectively; *p* < 0.05). Therefore, we speculated that FTO plays a major role as a core enzyme in placental m6A modification imbalance induced by fear stress.

### m6A Methylation Map in Placental Tissue Samples From Model and Control Rats

**FIGURE 4:**
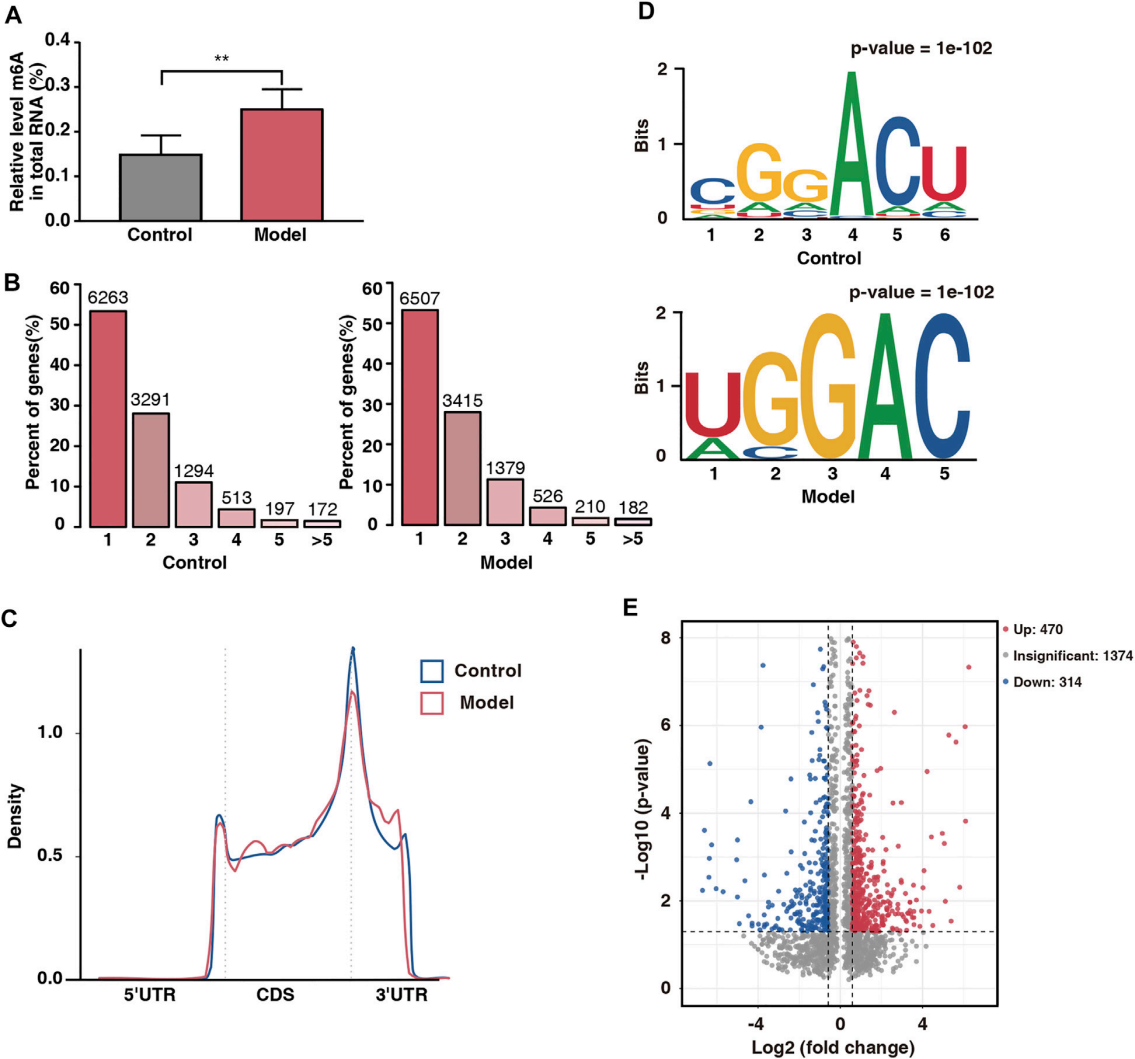
Overview of placental m6A methylation in fear stress during pregnancy. **(A)** m6A modification levels of total RNA in control and model groups (*n* ^*^ = 6, ^**^ *p* < 0.01). **(B)** Number of peaks of m6A-modified genes in control and model groups. Sequence motifs with m6A peak regions in control and model groups. **(C)** Density of differential m6A peaks along transcripts. **(D)** Sequence motifs with m6A peak regions in control and model groups. **(E)** Distribution of differentially modified m6A peaks in control and model groups (fold change ≥1.5 and *p* < 0.05).

Six placental tissue were randomly selected from each group to assess the overall level of m6A modification in the placenta. The levels of m6A modification in the model rats were significantly higher than those in the control rats (Figure 4A), suggesting that m6A modification is involved in placental damage caused by fear stress. Next, we selected three placental tissue per group for m6A-seq, and after pre-processing and quality control of the raw data, approximately 45 million valid data were obtained (Table 3). Of these data, 22,010 m6A peaks were associated with 12,219 gene transcripts in the model group, whereas 21,060 m6A peaks were associated with 11,730 gene transcripts in the control group. Further statistical analysis of the transcripts containing m6A modifications in both groups showed that the majority of the modified transcripts contained 1–3 m6A peaks (Figure 4B). To clarify the observed distribution of m6A sites on the transcriptome, m6A peak datasets were divided based on gene location. Specifically, m6A sites are mainly present in five regions: 5ʹ untranslated region (UTR), start codon, coding DNA sequence (CDS), stop codon, and 3ʹ UTR. We found that the m6A peaks were mainly located at the beginning of the CDS and 3ʹ UTR. This is consistent with the distribution of peaks previously found in mammalian systems. Interestingly, a unique m6A peak was found at the end of the 5ʹ UTR in our study (Figure 4C).

**TABLE 3:** Sequencing reads and alignment statistics in placental of control and model rats.

Sample_ID	Raw reads	Valid reads	Valid%	Q20%	Q30%	GC%
Control-1 IP	55148418	54034460	90.55	97.71	93.45	51.70
Control-1 input	50843246	50127664	90.96	97.91	93.83	51.07
Control-2 IP	55352890	54347860	91.13	97.68	93.35	52.25
Control-2 input	55089734	54266690	91.18	97.82	93.63	52.17
Control-3 IP	55978166	54924124	90.84	97.67	93.34	51.83
Control-3 input	52129688	51397122	91.12	97.83	93.64	51.42
Model-1 IP	46581006	45716122	91.14	97.65	93.25	51.19
Model-1 input	54677212	53943366	91.32	97.76	93.49	50.24
Model-2 IP	52450058	51449630	91.01	97.80	93.65	51.34
Model-2 input	53611946	52843856	91.37	97.79	93.59	50.78
Model-3 IP	49856318	48983724	91.26	97.75	93.48	52.07
Model-3 input	54761116	54051736	91.81	97.84	93.66	51.20

To determine whether the m6A peaks contained the classical m6A sequence RRACH (where R represents a purine, A represents m6A, and H represents a nonguanine base), we performed *de novo* motif analysis on the detected m6A sites and found a classical motif, the GGACU sequence, in both groups (Figure 4D). This finding strengthened the possibility of an m6A peak being present.

### GO and KEGG Analysis of Differentially Methylated mRNAs

To further analyze the abundance of m6A peaks in the two groups, we used exomePeak to screen 784 differential peaks at a fold change of ≥1.5 and a *p*-value of <0.05, including 470 hypermethylated sites and 314 hypomethylated sites (Figure 4E). The number of hypermethylated sites was significantly greater than that of hypomethylated sites, suggesting that fear stress increases placental m6A modification levels.

**FIGURE 5:**
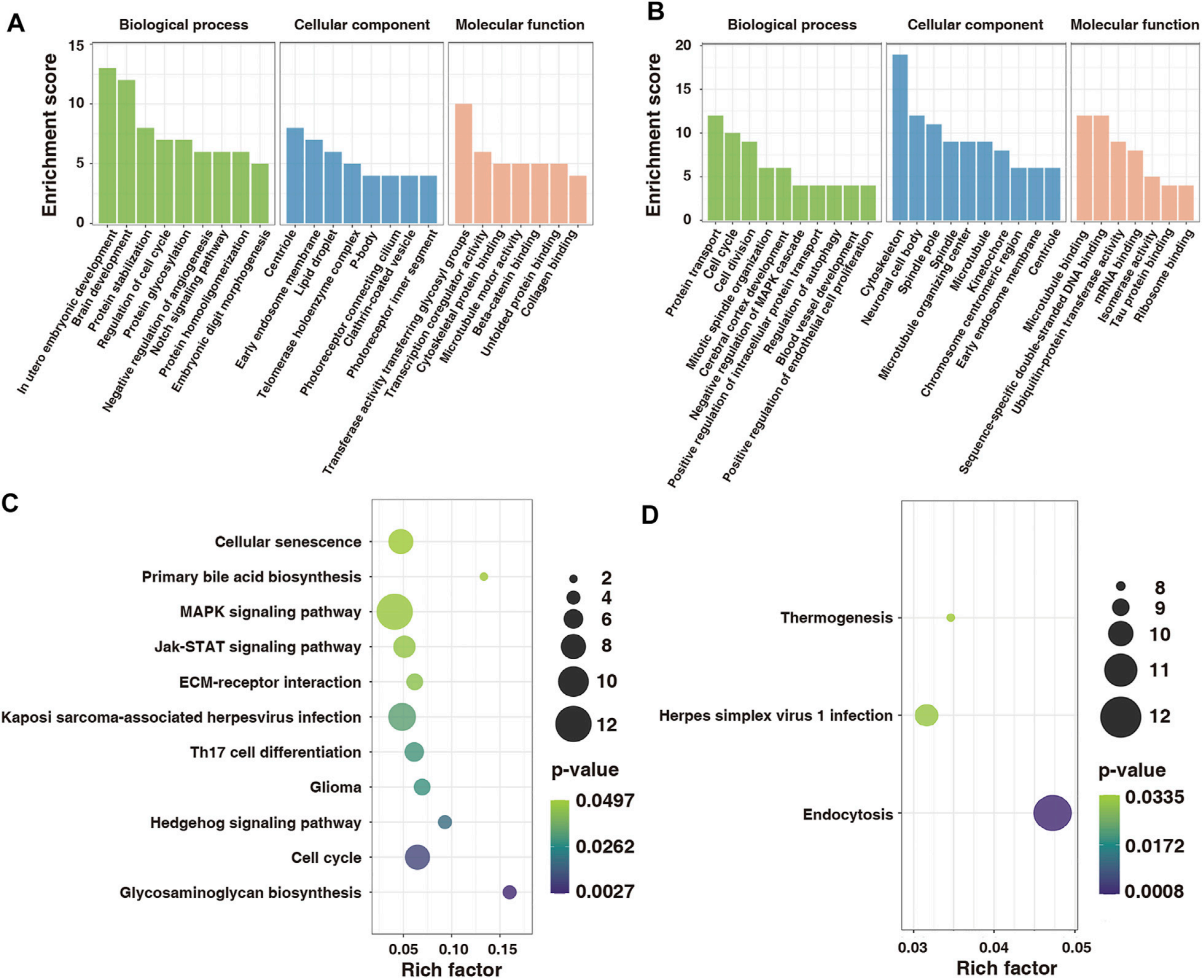
GO and KEGG functional annotation for differentially modified genes in control and model groups. **(A)** GO enrichment of hypermethylated genes. **(B)** GO enrichment of hypomethylated genes. **(C)** Enrichment of hypermethylated genes in the KEGG pathway. **(D)** Enrichment of hypomethylated genes in the KEGG pathway. GO, Gene Ontology; KEGG, Kyoto Encyclopedia of Genes and Genomes.

To explore the function of m6A-modified mRNAs, the GO function and KEGG pathway annotation of differentially methylated genes were analyzed. GO analysis revealed that the hypermethylated genes were significantly enriched *in utero* embryonic development, protein stabilization, negative regulation of angiogenesis, and embryonic digit morphogenesis (Figure 5A). In contrast, the hypomethylated genes were significantly enriched in protein transport, cell cycle, cell division, and positive regulation of intracellular protein transport (Figure 5B). KEGG pathway analysis showed that the hypermethylated genes were significantly correlated with the MAPK signaling pathway, Hedgehog signaling pathway, and cell cycle (Figure 5C), whereas the hypomethylated genes were related to endocytosis, herpes simplex virus one infection, and thermogenesis (Figure 5D).

### RNA-Seq and Combined Analysis of m6A MeRIP-Seq and RNA-Seq Results

**FIGURE 6:**
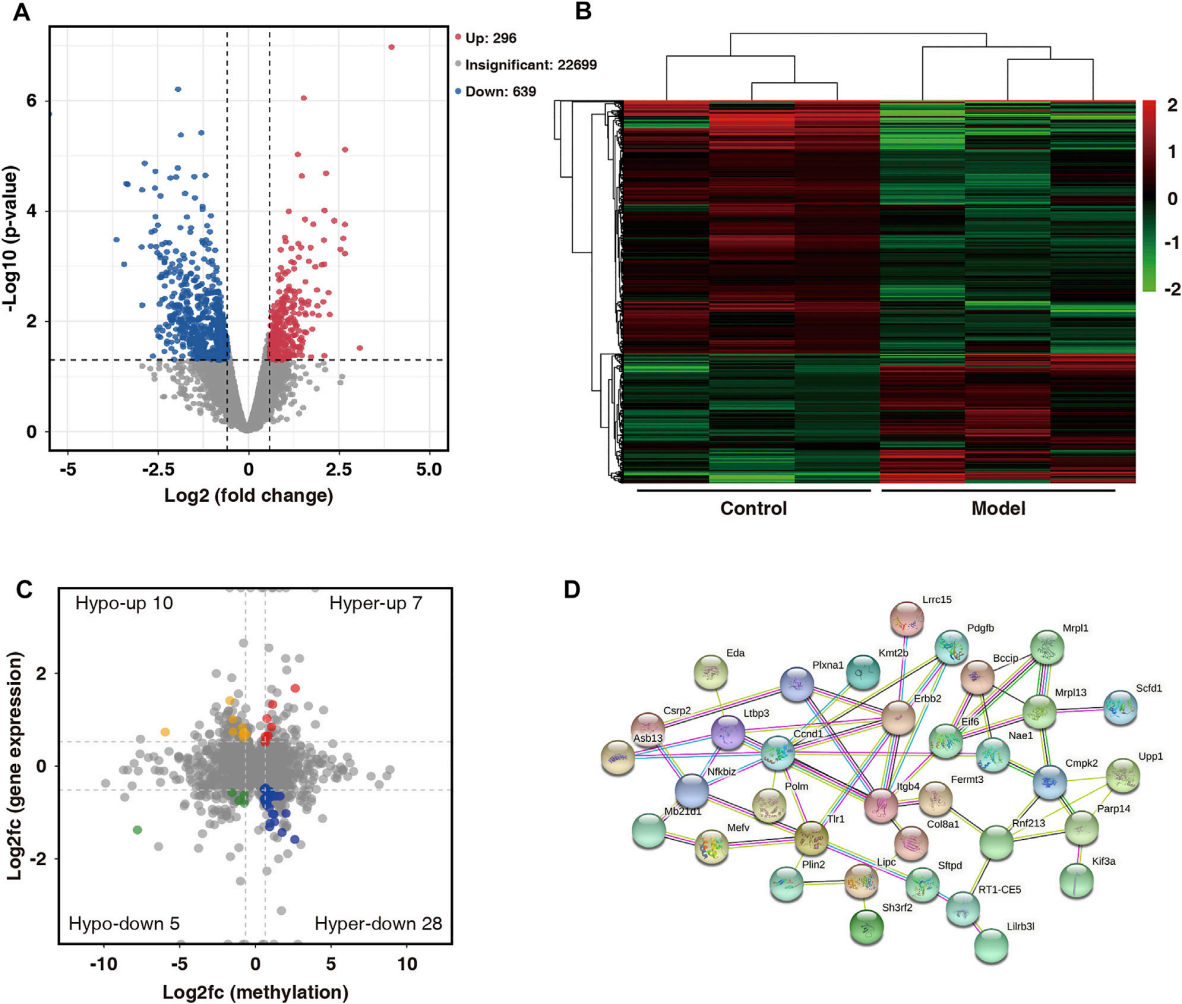
Combined analysis of MeRIP-seq and RNA-seq data for control and model group. **(A)** Distribution of differentially expressed mRNAs in control and model groups (fold change ≥1.5 and *p* < 0.05). **(B)** Hierarchical clustering analysis of differentially expressed mRNAs. **(C)** Differentially modified and differentially expressed genes (“double aberrant genes”) in control and model groups (fold change ≥1.5 and *p* < 0.05). **(D)** PPI network analysis of 50 double aberrant genes.

On comparing the RNA-seq data of the two groups, 935 differentially expressed genes (fold change ≥1.5 and *p* < 0.05) were found. Among them, 296 were upregulated and 639 were downregulated. The volcano map shows significantly different gene expressions between the two groups (Figure 6A). Subsequently, the 935 differentially expressed genes were used in hierarchical cluster analysis to identify differentially expressed genes in the two groups of samples (Figure 6B). By cross-analysis of the MeRIP-seq and RNA-seq data, 50 double aberrant genes were identified and divided into four groups: 7 “hyper-up” genes (highly methylated and overexpressed), 28 “hyper-down” genes (highly methylated and underexpressed), 10 “hypo-up” genes (hypomethylated and overexpressed), and 5 “hypo-down” genes (hypomethylated and underexpressed) (Figure 6C).

### Verification of m6A Methylation Status and Gene Expression of Specific Genes

**FIGURE 7:**
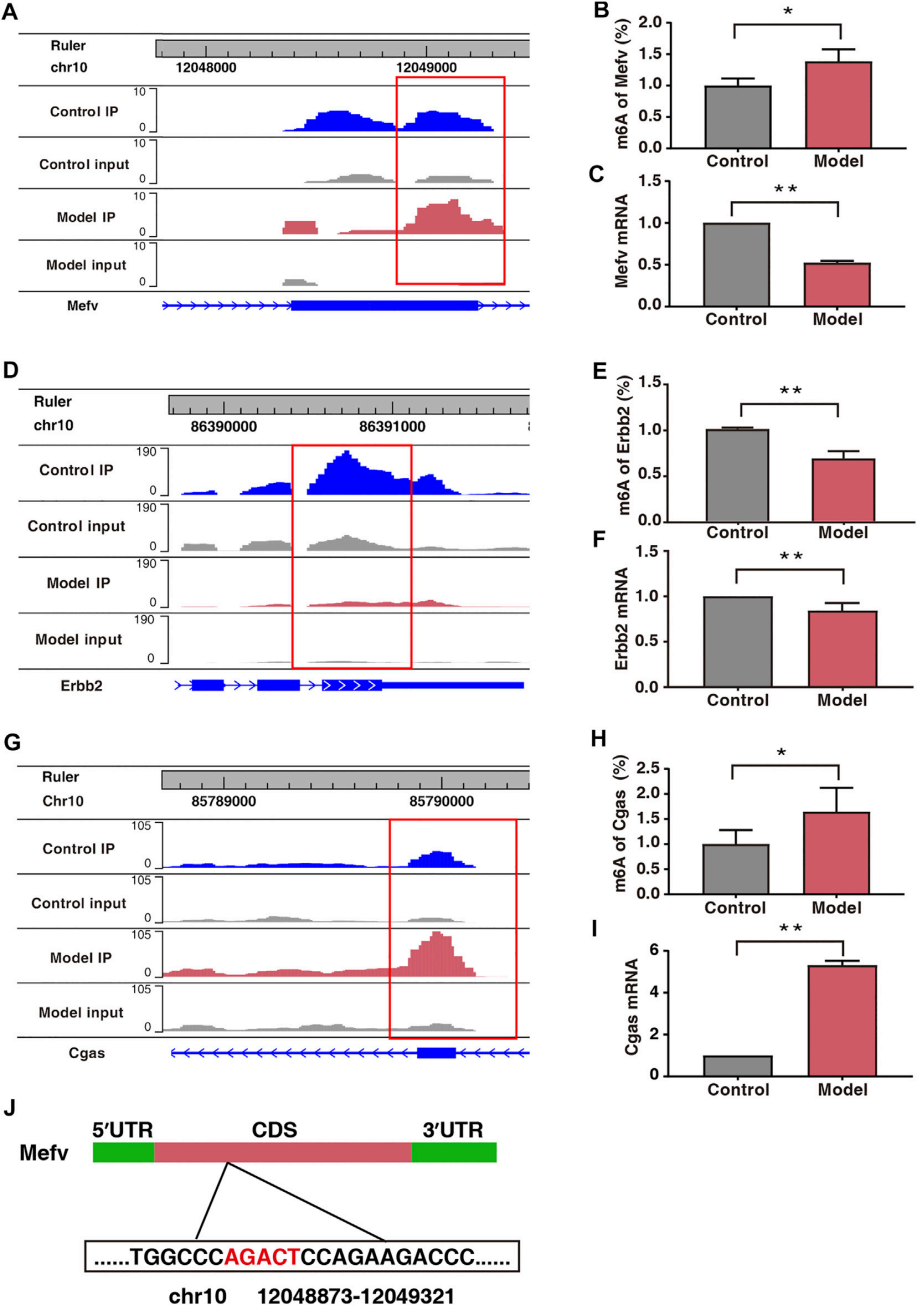
Expression of *Mefv*, *Erbb2*, and *Cgas* was regulated by m6A modification. **(A)** Data visualization analysis of *Mefv* mRNA m6A modification in the two groups. **(B)** m6A modification levels of *Mefv* at a particular site (*n* ^*^ = 6, ^**^ *p* < 0.05). **(C)** mRNA levels of *Mefv* in the two groups (*n* ^*^ = 6, ^**^ *p* < 0.01). **(D)** Data visualization analysis of *Erbb2* mRNA m6A modification in the two groups. **(E)** m6A modification levels of *Erbb2* at a particular site (*n* ^*^ = 6, ^**^ *p* < 0.01). **(F)** mRNA levels of *Erbb2* in the two groups (*n* ^*^ = 6, ^**^ *p* < 0.01). **(G)** Data visualization analysis of *Cgas* mRNA m6A modification in the two groups. **(H)** m6A modification levels of *Cgas* at a particular site (*n* ^*^ = 6, ^**^ *p* < 0.05). **(I)** mRNA levels of *Cgas* in the two groups (*n* ^*^ = 6, ^**^ *p* < 0.01). **(J)** Schematic representation of the location of m6A modification site in *Mefv*.

Protein–protein interaction (PPI) network analysis was performed on 50 double aberrant genes. A total of 27 double aberrant genes were found to have two or more nodes (Figure 6D; Table 4). *Mefv* and *Erbb2*, the highest and lowest node genes, were selected according to fold change. In addition, to confirm the accuracy of the sequencing results, we selected the non-node gene *Cgas* for verification experiments. MeRIP-qPCR and RT-qPCR were used to verify the m6A modification and mRNA expression of the three genes. *Mefv* showed hypermethylation and downregulated mRNA expression (Figures 7A–C), *Erbb2* showed hypomethylation and downregulated mRNA expression (Figures 7D–F), and *Cgas* showed hypermethylation and upregulated mRNA expression (Figures 7G–I). These results were consistent with the sequencing data.

**TABLE 4:** Double aberrant genes in PPI network.

Chromosome	Gene name	Peak start	Peak end	log2 (fold change)	*p*	m6A modification level
chr10	*Mefv*	12048873	12049321	2.6	0.01	up
chr11	*Col8a1*	45004814	45005172	2.02	0.02	up
chr14	*Tlr1*	45064237	45064564	1.76	0.01	up
chr11	*Parp14*	68135559	68136457	1.62	0.00	up
chr8	*Lipc*	77272600	77276649	1.14	0.03	up
chr1	*Fermt3*	222271705	222272285	1.13	0.01	up
chr4	*Plxna1*	121217659	121217809	1.13	0.00	up
chr20	*RT1-CE5*	4896430	4896991	1.05	0.01	up
chr7	*Pdgfb*	121214957	121215137	0.997	0.00	up
chr6	*Cmpk2*	45694642	45694822	0.94	0.00	up
chr5	*Plin2*	104984414	104984594	0.924	0.00	up
chr10	*Itgb4*	104560303	104560362	0.74	0.03	up
chr1	*Kmt2b*	89037521	89038195	0.673	0.00	up
chr1	*Ccnd1*	218090750	218091018	0.661	0.00	up
chr16	*Sftpd*	18753673	18753823	0.643	0.00	up
chr1	*Ltbp3*	221115887	221116096	0.59	0.00	up
chr3	*Eif6*	151356868	151356985	0.586	0.00	up
chr7	*Mrpl13*	95288406	95293667	−0.611	0.00	down
chr11	*Nfkbiz*	47270325	47270564	−0.741	0.00	down
chr1	*Bccip*	205777455	205783608	−0.742	0.00	down
chr7	*Csrp2*	53630621	53630680	−0.773	0.01	down
chr17	*Asb13*	70216354	70217243	−0.859	0.01	down
chr10	*Rnf213*	108579105	108579315	−1.1	0.03	down
chr19	*Nae1*	653510	659518	−1.47	0.00	down
chr14	*Mrpl1*	14982671	15008090	−1.49	0.00	down
chr14	*Upp1*	89314266	89314446	−1.54	0.02	down
chr10	*Erbb2*	86390165	86390803	−7.78	0.00	down

## Discussion

In this study, we performed the high-throughput sequencing of the placenta obtained from rat models of fear stress and found that fear stress during pregnancy can alter the levels of four m6A enzymes (METTL3, METTL14, WTAP, and FTO) as well as the levels of methylation modifications in the placenta, thereby revealing a specific m6A methylation profile for placenta with injury induced by fear stress during pregnancy. Some double aberrant genes were also found, which may be related to the regulation of placental function. Therefore, we infer that m6A modifications may regulate the expression levels of these genes and may play an essential role in the way fear stress response affects placental development.

Fear, a common psychological stress, is unlikely to cause disease in a short time or at low intensity. Nevertheless, long-term fear stimulation beyond physical adaptability and tolerance will lead to stress injury. Our group’s previous research found that fear stress can lead to placental dysfunction and adverse fetal outcomes. The placenta has always been regarded as a critical link between the mother and fetus. It is well known that placental damage can lead to adverse fetal outcomes (Sun et al., 2020). In this study, we found that fear stress can reduce the number of live births, the total weight of the placenta, and the weight of the individual placenta.

M6A modification is a dynamic and reversible process that is involved in a variety of biological processes. Not only can affect mRNAs encoding histone modifiers and transcription factors but also chromatin-associated regulatory RNAs (Wei and He, 2021). Many studies related to m6A have been conducted in mammals, plants, and yeast. Previous studies in animals have shown that fear stress can modulate the expression of methylase in different regions of the brain, and can thereby affect m6A modification levels. Exposure to specific stressors and glucocorticoid levels can alter METTL3 and FTO expression in adult neurons, thereby affecting m6A modification levels and ultimately causing synaptic plasticity and increasing fear memory. Fear stress also decreased FTO levels and increased m6A modification levels in the neurons of the dorsal CA1 region of the hippocampus (Walters et al., 2017). Knocking down FTO in the medial prefrontal cortex of mice was found to increase m6A modification levels, which was associated with increased fear memory in mice (Widagdo et al., 2016).

However, there is a knowledge gap in regards to whether fear stress can alter methylation levels and methylesterase expression in the placenta. This study showed that fear stress during pregnancy can increase the overall levels of m6A modification in the placenta and alter the expression levels of METTL3, METTL14, WTAP, and FTO in the placenta. This critical finding provides an entry point for future studies on the relationship between epigenetic modifications and placental dysplasia induced by fear stress.

The specific increase in m6A modification levels in the placentas of rat models of fear stress may be associated with the altered expression of the methylation enzymes METTL3, METTL14, WTAP, and FTO. METTL3 is a core component of the catalytic subunit and is involved in cell development, cellular homeostasis, and cellular recoding (Liu et al., 2014). The primary role of METTL14 is to expand the range of RNA substrate recognition and enhance catalytic efficiency (Wang et al., 2016). METTL3 and METTL14 play essential roles in placental development and function. In the placentas of patients with eclampsia, both METTL3 and METTL14 are upregulated. METTL3 prevents trophoblast proliferation, migration, and invasion by promoting the recognition of pri-miR-497-5p/195-5p by DGCR8 (Wang J. et al., 2020). In our study, the expression levels of METTL3, METTL14, and WTAP in the placentas of rat models of fear stress were significantly increased, which corroborated the findings of the previously mentioned study. FTO was the first enzyme found to have an efficient oxidative demethylation activity (Jia et al., 2011), and its functional role in regulating placental and fetal development has received widespread attention. A study revealed that FTO expression was negatively correlated with placental quality in primiparous pregnancies and positively correlated with fetal weight and length (Bassols et al., 2010). Another study found that elevated FTO expression levels in the placenta were associated with an increased transport capacity of specific amino acid transport proteins, thereby ensuring efficient nutrient transport and promoting faster head size growth and increased fetal length in newborns (Barton et al., 2016). Notably, FTO expression was reduced and m6A modification was increased in the placental tissues of piglets with low birth weight; this was theorized to affect the expression of angiogenic and lipid metabolism genes in the placenta (Song et al., 2018). FTO knockout female mice exhibit significant ovarian defects and impaired fertility, which is associated with the involvement of m6A modification in regulating oocyte maturation and embryonic development. The absence of FTO caused increased m6A levels of LINE1 and decreased RNA levels of LINE1 in oocytes and embryos, while further indicating that the FTO-LINE1 RNA axis is functionally relevant in oocyte and embryo development in the mouse. This observation also indicates the prominent role of FTO-mediated m6A modifications in early embryonic development (Wei et al., 2022). In our study, the expression of placental FTO was significantly decreased due to fear stress. Correlation analysis showed that the expression of FTO was positively correlated with fear in pregnant rats and negatively correlated with the number of live births and the weight of the placenta, suggesting that FTO plays a major role as a core regulator of the imbalance in placental m6A modification caused by fear stress during pregnancy.

The RRACH motif is a classical sequence involved in m6A modification (Tao et al., 2017; Fitzsimmons and Batista, 2019), and from our experimental results, a common GGACU sequence was identified in both groups. This also supports the plausibility that m6A modifications occur in both groups. To investigate the potential functions of m6A-modified genes, GO and KEGG enrichment of differentially methylated peaks were performed. The results of GO enrichment revealed that the m6A-modified genes were mainly associated with angiogenesis, vascular endothelial cell development, protein transport, and cell cycle. The results of KEGG analysis revealed that the m6A-modified genes were mainly enriched in the MAPK signing pathway, Hedgehog signing pathway, and other signaling pathways. The MAPK signing pathway, a classical pathway, is involved in the regulation of embryonic development, placental development, vascular development, and other biological processes. In our study, the 12 aberrantly modified genes induced by fear stress were enriched in this pathway. It was previously reported that GPR4 can mediate oocyte maturation and regulate trophoblast infiltration and invasion via MAPK signaling (Qi et al., 2021). Moreover, PGF2α can reportedly promote angiogenesis in the porcine endometrium by activating the MAPK signaling pathway (Kaczynski et al., 2020). The overexpression of ERBB4 can also enhance vascular development via MAPK signaling (Liang et al., 2019). The Hedgehog signing pathway, which is involved in the induction of placental endoderm cell production, is closely associated with placental development and function and has also been prominently implicated in the etiology of PE (Jiang and Herman, 2006; Huang et al., 2021). The downregulation of the Hedgehog signaling pathway inhibits translation in the epithelial mesenchyme, attenuates trophoblast invasion and migration, and induces PE (Chen et al., 2019). In our study, genes with differential m6A modification were significantly enriched in these two pathways. Thus, m6A modification may regulate gene expression via the MAPK and Hedgehog signaling pathways. Further validation of these two pathways could help reveal new gene regulation mechanisms by which fear stress affects placental development at the mRNA level.

In our study, 50 double aberrant genes were identified by combining MeRIP-seq and RNA-seq data. These may be the target genes involved in placental m6A modification imbalance caused by fear stress. Three genes, *Mefv*, *Erbb2*, and *Cgas*, were selected for further analysis based on String results and in combination with low *p* and high FC values. We found elevated m6A modification levels and decreased mRNA expression levels of *Mefv* in the placental tissues of model rats. The decreased expression of *Mefv* can be attributed to the degradation function of m6A modification. Moreover, the reader proteins YTHDF1-3, FMR1, and HNRNPA2B1 were found in the core of mammalian stress granules (Jain et al., 2016). Recently, it has been found that the binding protein FMR1 can preferentially bind to mRNA containing m6A marker and “AGACU” motif and participate in the degradation of target mRNA by utilizing m6A modification (Zhang et al., 2022). In this study, RNA-seq analysis revealed that the binding protein FMR1 was significantly expressed in the placental tissues of model rats, and further sequence resolution of the m6A-modified region of *Mefv* revealed the sequence “AGACT (T = U)” (Figure 7J; Table 5). Therefore, we believe that FMR1 recognizes the upregulation of m6A modification induced by fear stress, thereby triggering degradation of its mRNA and ultimately leading to reduced expression of *Mefv*. Abnormal changes in *Mefv* can cause familial Mediterranean fever (FMF) (Grandemange et al., 2011; Kirectepe et al., 2011), and there has been extensive research on *Mefv* in FMF. However, there remains a gap in research on stress-induced placental injury. This study reported that the function of *Mefv* may not be limited to FMF. *Erbb2* was another node gene whose m6A modification and mRNA expression levels decreased significantly. The target binding protein of Erbb2 was not determined in this study. Erbb2 is an epidermal growth factor receptor family member (Niu and Carter, 2007; Sharma et al., 2021), and its upregulation can increase angiogenesis. Erbb2 is also closely related to many genes related to vascular development in the genome. The overexpression of Erbb2 inhibits the transcription of antiangiogenic factors (Sparc, Timp3, and Serpinf1) but induces the expression of angiogenic factors (Klf5, Tnfaip2, and Sema3c) (Beckers et al., 2005). However, Erbb2 has not been reported to be associated with placental injury induced by fear stress during pregnancy.

**TABLE 5:** Expression of m6A methylation regulator.

Gene name	Chromosome	log2 (fold change)	*p*	Expression level
*Ythdf3*	chr2	0.25	0.39	up
*Ythdf1*	chr3	−0.24	0.43	down
*Ythdc2*	chr1	0.17	0.73	up
*Ythdf2*	chr5	0.05	0.77	up
*Ythdc1*	chr14	−0.06	0.98	down
*Fmr1*	X	0.95	0.00	up
*Lrpprc*	chr6	−0.25	0.42	down
*Igf2bp1*	chr10	−0.28	0.50	down
*Alyref*	chr10	−0.21	0.55	down
*Elavl1*	chr12	0.10	0.61	up

In this study, *Mefv* and *Erbb2* were abnormally methylated and differentially expressed in the placentas of rat models of fear stress. PPI network analysis also showed that *Mefv* and *Erbb2* were involved in regulating several gene clusters. Therefore, we speculated that these two genes have excellent research potential as the target genes of fear stress that alter m6A modification in the placenta and lead to placental injury.

**FIGURE 8:**
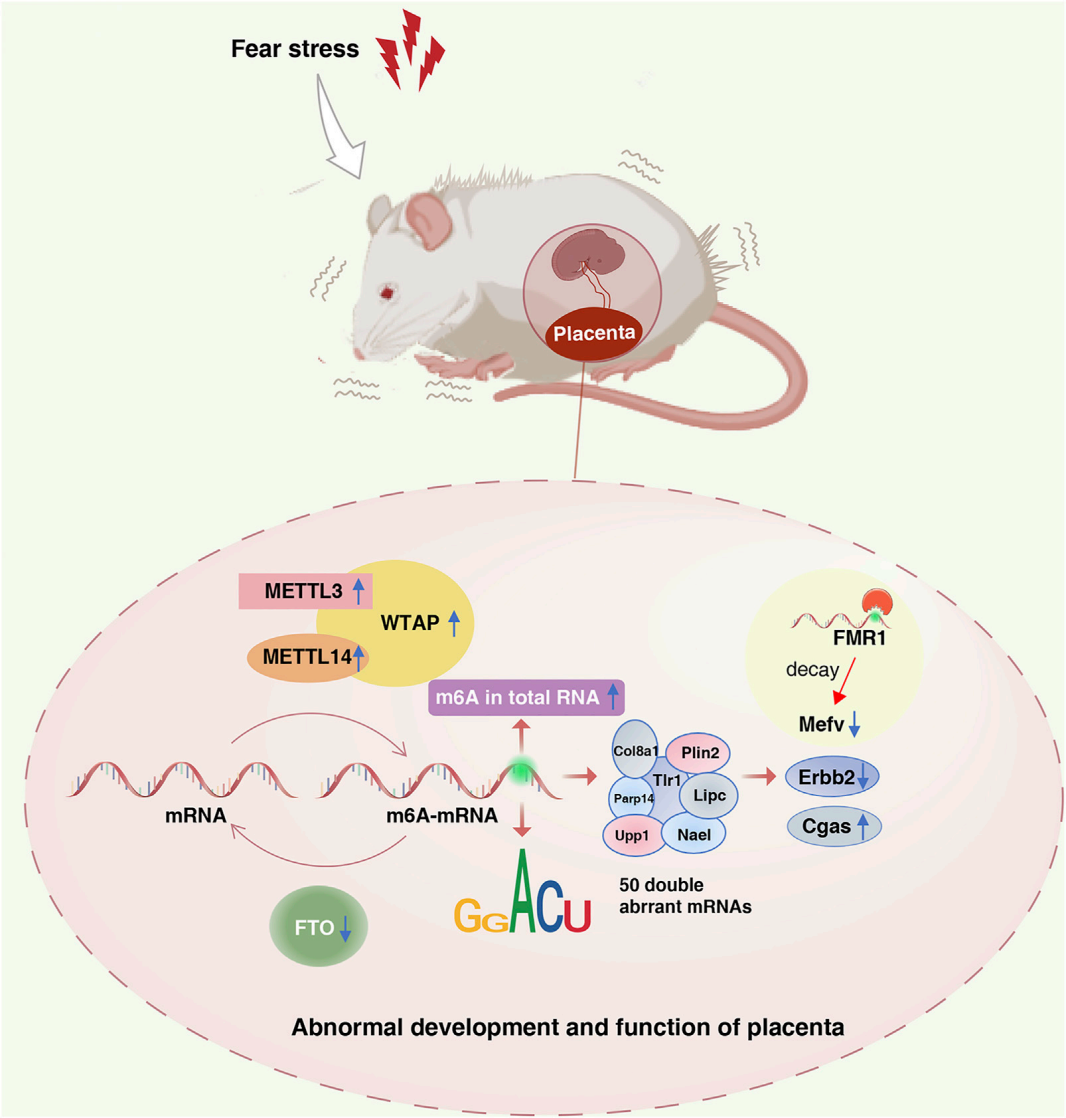
Role of the m6A modification in placental dysfunction induced by fear stress during pregnancy. Fear stress during pregnancy increases the levels of methylated enzymes (METTL3, METTL14, and WTAP), decreases the levels of demethylase FTO, and increases the overall methylation levels in the placenta of pregnant rats. Fear stress during pregnancy can lead to abnormal modification and differential expression of 50 genes. Decreased expression of Mefv may rely on the decay function of m6A modific.

In summary, this study analyzed the effects of fear stress on enzyme methylation and methylation modification levels in the placenta, suggesting that m6A modification plays a role in placental dysfunction induced by fear stress during pregnancy (Figure 8). MeRIP-seq was used to sequence the placental tissues, providing a basis for revealing the functional mechanism of placental injury caused by fear stress during pregnancy based on m6A modification levels. Nevertheless, there are some limitations of our study. We determined that fear stress during pregnancy affects the expression of methylase in the placenta and preliminarily identified some differentially expressed genes. However, as we did not perform targeted inhibition or activation of specific methylases, we cannot determine the specific function of each enzyme. In the future, we plan on exploring this aspect more and investigating this topic in greater depth.

## Conclusion

This was the first study to investigate changes in placental m6A-modifying enzymes and modification levels causing adverse fetal outcomes due to fear stress during pregnancy. Fifty double aberrant genes were identified by comprehensive MeRIP-seq and RNA-seq data analyses. *Mefv*, *Erbb2*, and *Cgas* were selected for validation, and the validation results were consistent with the sequencing data. We speculate that an imbalance in placental m6A modification is one of the potential pathogenic mechanisms by which fear stress during pregnancy causes placental damage. These double aberrant genes may be the target genes of placental dysfunction caused by fear stress during pregnancy.

## Data Availability

The datasets presented in this study can be found in online repositories. The names of the repository/repositories and accession number(s) can be found below: https://www.ncbi.nlm.nih.gov/bioproject/PRJNA837736.
